# Pain Assessment in the Emergency Department: A Prospective Videotaped Study

**DOI:** 10.5811/westjem.2022.6.55553

**Published:** 2022-08-28

**Authors:** Hao-Ping Hsu, Ming-Tai Cheng, Tsung-Chien Lu, Yun Chang Chen, Edward Che-Wei Liao, Chih-Wei Sung, Chiat Qiao Liew, Dean-An Ling, Chia-Hsin Ko, Nai-Wen Ku, Li-Chen Fu, Chien-Hua Huang, Chu-Lin Tsai

**Affiliations:** * National Taiwan University, College of Medicine, Department of Medicine, Taipei, Taiwan; † National Taiwan University Hospital, Department of Emergency Medicine, Taipei, Taiwan; ‡ National Taiwan University, College of Medicine, Department of Emergency Medicine, Taipei, Taiwan; § National Taiwan University Hospital Yun-Lin Branch, Department of Emergency Medicine, Hsinchu, Taiwan; ¶ National Taiwan University Hospital Hsin-Chu Branch, Department of Emergency Medicine, Hsinchu, Taiwan; || National Taiwan University, Department of Computer Science and Information Engineering, Taipei, Taiwan

## Abstract

**Introduction:**

Research suggests that pain assessment involves a complex interaction between patients and clinicians. We sought to assess the agreement between pain scores reported by the patients themselves and the clinician’s perception of a patient’s pain in the emergency department (ED). In addition, we attempted to identify patient and physician factors that lead to greater discrepancies in pain assessment.

**Methods:**

We conducted a prospective observational study in the ED of a tertiary academic medical center. Using a standard protocol, trained research personnel prospectively enrolled adult patients who presented to the ED. The entire triage process was recorded, and triage data were collected. Pain scores were obtained from patients on a numeric rating scale of 0 to 10. Five physician raters provided their perception of pain ratings after reviewing videos.

**Results:**

A total of 279 patients were enrolled. The mean age was 53 years. There were 141 (50.5%) female patients. The median self-reported pain score was 4 (interquartile range 0–6). There was a moderately positive correlation between self-reported pain scores and physician ratings of pain (correlation coefficient, 0.46; P <0.001), with a weighted kappa coefficient of 0.39. Some discrepancies were noted: 102 (37%) patients were rated at a much lower pain score, whereas 52 (19%) patients were given a much higher pain score from physician review. The distributions of chief complaints were different between the two groups. Physician raters tended to provide lower pain scores to younger (P = 0.02) and less ill patients (P = 0.008). Additionally, attending-level physician raters were more likely to provide a higher pain score than resident-level raters (P <0.001).

**Conclusion:**

Patients’ self-reported pain scores correlate positively with the pain score provided by physicians, with only a moderate agreement between the two. Under- and over-estimations of pain in ED patients occur in different clinical scenarios. Pain assessment in the ED should consider both patient and physician factors.

## INTRODUCTION

Acute pain is one of the most common complaints of patients presenting to the emergency department (ED).^1^ Pain score is a valid and reliable tool to assess pain and may lead to better pain management.^2^,^3^ Both visual analog scales and numeric rating scales (NRS) are considered appropriate measurements of self-reported pain in the ED.^4^ Some professional societies suggest that pain could and should be measured as a biologic metric akin to other vital signs.^5^ However, the notion of pain assessment at all clinical encounters was not universally supported by medical professionals, as some studies have shown no significant improvement in pain management associated with pain measurements.^6^, ^7^

Inappropriate pain management, such as oligoanalgesia, remains common in the ED.^8^,^9^ Oligoanalgesia could be attributed to many reasons, one of which is the underappreciation of patient self-reported pain by healthcare professionals, leading to fewer pain medications.^7^,^10^ Although self-reported pain scores are traditionally viewed as the “gold standard” in pain assessment, some research studies suggest this simplistic approach ignores the complex relationship between patients and clinicians.^11^,^12^ Instead, pain assessment should be regarded as a social transaction between patients and clinicians.^13^ Despite the potentially complex construct underlying pain assessment, few studies have attempted to evaluate the agreement between patient self-reported pain and physicians’ perception of patient pain in the ED. In addition, little is known regarding factors associated with the discrepancy between the two approaches. Addressing these knowledge gaps may lead to a better and more holistic understanding of pain assessment.

Therefore, in this prospective study we sought to assess the agreement between pain scores reported by patients and those gauged by physicians. In addition, we attempted to identify patient and physician factors that lead to greater discrepancies in pain assessment.

## METHODS

### Study Design and Setting

From May 2020–January 2021, we conducted a prospective observational study in the ED of the National Taiwan University Hospital (NTUH). The NTUH is a tertiary academic medical center with approximately 2,400 beds and 100,000 ED visits per year. Trained research personnel prospectively enrolled patients who presented to the ED using a standard protocol. Inclusion criteria were age ≥20 years (legal age of majority in Taiwan) and the ability to provide informed consent. We excluded patients who needed immediate cardiopulmonary resuscitation, those with psychiatric complaints or consciousness disturbance, or those who needed isolation for infection control. A high-sensitivity camera and a clip-on Bluetooth microphone were set up to record the entire triage process, including patient facial images and conversations between patients and triage nurses (online Supplementary eFigure).

**Population Health Research Capsule**

What do we already know about this issue?

*Research suggests that pain assessment involves a complex interaction between patients and clinicians*.

What was the research question?

*We measured the agreement between pain scores reported by patients and pain assessed by emergency physicians via video review*.

What was the major finding of the study?

*Patients’ self-reported pain scores correlated positively with the pain score provided by physicians (correlation coefficient 0.46, kappa 0.39), with only a moderate agreement*.

How does this improve population health?

*Under- and over-estimations of pain in ED patients occur in different clinical scenarios. Pain assessment in the ED should encompass both patient and physician factors*.

### Measurements

In Taiwan, ED triage is conducted by senior ED triage nurses who are familiar with a computerized triage software called the Taiwan Triage and Acuity Scale (TTAS). The TTAS was adapted from the Canadian Triage and Acuity Scale (CTAS) and has been validated against hospitalization, length of ED stay, and resource utilization.^14^ The TTAS requires the input of pain scores on a NRS of 0 to 10, with 0 being no pain and 10 being the worst pain imaginable. Pain scores were directly solicited from patients unless they were not able to report it themselves. We further categorized the NRS scores into no (0), mild (1–3), moderate (4–6), and severe (7–10) pain.^15^

We also retrieved the computerized TTAS system that contains information on a total of 179 structured chief complaints. Based on the computerized algorithms, the TTAS classifies patients in the following order of acuity: level 1, resuscitation; level 2, emergent; level 3, urgent; level 4, less urgent; and level 5, non-urgent. Other triage data were collected, including demographics, mode of arrival, trauma mechanisms, work-related injury, past medical history, structured chief complaints, vital signs (temperature, heart rate, systolic and diastolic blood pressure, respiratory rate, oxygen saturation), body weight, height, and levels of consciousness coded per the Glasgow Coma Scale (GCS).

### Video Data and Physician Review

The recorded videos underwent quality checks to ensure adequate sound and image quality. Five emergency physician reviewers (three senior residents and two attending physicians) were recruited and trained via educational meetings. Reviewers were provided with triage electronic health records but were blinded to the pain score documented; however, they may have overheard self-reported pain scores during the video review. Reviewers were asked to provide their perceived pain scores based on not only self-reported pain scores, but also objective clues, including chief complaints, facial expressions, body posture, vocalization, and vital signs.^16^–^18^ The physician-perceived pain scores were also rated on a NRS of 0 to 10, with 0 being no pain and 10 being the worst pain. The first five videos served as pilot data (four women and one man; mean age 67 years) and were rated by each reviewer. The intraclass correlation coefficient (ICC) that quantified the inter-observer agreement on perceived pain scoring between reviewers reached 0.59 for the pilot data. Afterward, the physician reviewers independently rated video recordings. Periodic investigator consensus meetings were held to discuss and resolve pain scoring issues.

This study was approved by the NTUH Institutional Review Board, and informed consent was obtained from all participants.

### Statistical Analysis

Summary statistics are presented as proportions (with 95% confidence intervals [CI]), means (with SD), or medians (with interquartile ranges [IQRs]). We examined bivariate associations using Student’s t-tests, Mann-Whitney tests, Fisher’s exact tests, and chi-square tests, as appropriate. The agreement of pain scoring was measured by the kappa statistic with quadratic weighting. We also used the ICC and Spearman’s correlation. A Bland-Altman plot was performed to assess the agreement of pain scoring between patient self-report and physician ratings of pain. We used a two-way scatterplot to depict the relationship between the two scoring approaches with a best-fit linear regression line.

Previous studies have shown that an approximately 1.30- to 1.65-point is the minimal clinically important difference (MCID) in the NRS from 0–10.^19^–^21^ As such, for this study we defined a ≥2-point difference in pain score as a significant discrepancy. Patients were then divided into two groups: group A with a significantly (≥2 points) lower pain rating from physician reviewers and group B with a significantly (≥2 points) higher pain rating from physician reviewers. A subset of group B (termed a vague-pain or suffering group) consisted of patients with a self-reported pain score of zero but received at least 2 points in pain score from physician reviewers.

We anticipated that the mean of differences between self-reports and physician ratings would be 0.5 and the SD of differences would be 0.65.^19^–^21^ Using the sample size calculation for assessing agreement between the two methods with an MCID of 2, a two-sided alpha of 0.05, and 90% power, we estimated that 259 subjects would need to be enrolled.^22^ We performed all analyses using Stata 16.0 software (StataCorp, College Station, TX). All *P*-values are two-sided, with *P* <0.05 considered statistically significant.

## RESULTS

**Figure 1:**
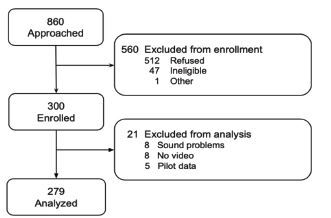
Flow diagram of patient selection process for study comparing patient and physician pain scoring at emergency department triage.

Figure 1 shows the patient selection process. In total, 860 patients were approached, and 560 patients were excluded owing to refusal to participate or ineligibility (age <20 years, psychiatric complaints, and consciousness disturbance). Among 300 enrolled patients, 16 patients were excluded because of video or sound issues, and five patients were used as pilot data. Overall, 279 patients were included in the final analysis.

Table 1 presents the clinical characteristics of patients. The mean age of the patients was 53.3 years, and 138 patients (50%) were male. All patients were Asian. The median self-reported pain score was 4 (IQR 0–6). A total of 125 patients (45%) reported no pain, 14 patients (5%) reported mild pain, 92 patients (33%) reported moderate pain, and 48 patients (17%) reported severe pain. Most patients were triaged to level 3, and the triage duration was about 2–3 minutes. Trauma/injuries (14%), abdominal pain (11%), fever (8%), dizziness and vertigo (8%), and chest pain (7%) were the top five most common chief complaints.

**Figure 2:**
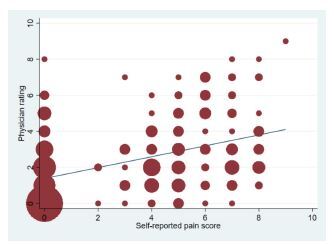
The scatterplot of self-reported pain scores and physicians’ ratings of pain. The line indicates the best-fit linear regression line. The sizes of circles are proportional to the number of observations.

Figure 2 represents the scatterplot of self-reported pain scores and physician ratings of pain. The relationship between the two approaches appeared to be positive (regression coefficient = 0.30; 95% CI 0.23–0.38, *P* < 0.001). There was a 0.3-point increase in physician rating per 1-unit increase in self-reported pain score. The correlation coefficient also showed a moderately positive correlation (0.46, *P* < 0.001). The ICC between the two scoring systems was 0.55. Table 2 shows the cross-tabulation of the two scoring systems. The weighted kappa coefficient was 0.39, suggesting a moderate agreement.

**Figure 3:**
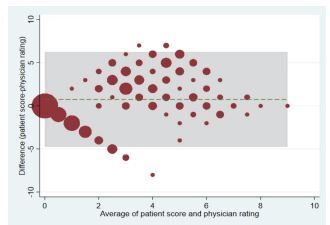
The Bland-Altman plot of the agreement between self-reported pain scores and physicians’ ratings of pain. The green line represents the mean difference between the patient and physician scores. The shaded box is bounded by the statistical limits of agreement (defined as the mean difference ± 1.96 SD of differences).

Figure 3 depicts the Bland-Altman plot of the physician- and self-reported pain scores. The green line represents the mean difference between the patient and physician scores (0.74; 95% CI 0.41–1.07). The green line was slightly above zero, indicating that patients rated their pain slightly higher than physicians’ perception. Some degree of disagreement was noted, as indicated by data points ≥ the two-point MCID or even beyond the statistical limits of agreement (ie, outside the shaded box). Most of the disagreements occurred in the middle range (moderate pain).

Among the 279 patients, 102 patients were rated at a much lower pain score (group A), whereas 52 patients were given a much higher pain score (group B). The baseline characteristics of patient groups A and B are listed in Table 3. Physician raters tended to give lower pain scores to younger and less ill (ie, lower triage levels) patients. We detected no differences in patient gender between the two groups. The distributions of chief complaints were quite different between the two groups. The most common chief complaints in group A were injuries (24%), abdominal pain (20%), soft tissue redness and swelling (11%), and chest pain (10%). In contrast, the most frequent chief complaints in group B were dizziness and vertigo (19%), fever (10%), and nausea and vomiting (8%). Regarding the rater-level influences, resident-level physician raters were more likely to give a lower pain score in group A. In contrast, attending-level physician raters were more likely to provide a higher pain score in group B.

In a subgroup analysis of group B, 49 patients were considered to have vague pain (Table 4). The most common chief complaints included dizziness and vertigo (18%), fever (10%), nausea and vomiting (8%), general weakness (6%), or injuries (6%). The median score given by the physician raters was 3 (IQR 2–4).

## DISCUSSION

In this prospective videotaped study, we found a moderate agreement between pain scores reported by patients and those given by physicians. In addition, physician raters tended to give lower pain scores to younger patients and patients with a lower triage level. By contrast, attending-level physician raters were more likely to provide a higher pain score, particularly for those suffering from illnesses not directly related to pain.

For group A, the results revealed that many patients reported higher pain scores than those based on physicians’ evaluations, a finding that is consistent with previous reports.^23^, ^24^ A common explanation for this discrepancy is that healthcare professionals frequently assess pain based on their experience rather than patients’ feelings.^25^, ^26^ Therefore, physicians tend to give lower pain scores when considering chief complaints that they thought were not that painful (eg, cellulitis).^24^ On the other hand, previous studies have demonstrated that patients tended to report inconsistent pain scores to nurses and treating physicians. For example, patients with foot and ankle problems reported higher pain scores to the surgeon than those to the nurse, perhaps to justify the urgency of their problems and receive quicker treatments. 27, 28 Alternatively, ED patients, especially those suffering from pain, might be anxious regarding their problems and hence were unable to gauge their painful feelings precisely.^13^ Taken together, it is prudent to evaluate pain not solely based on self-reported pain scores, which could be an overestimation and potentially lead to unnecessary analgesics.^29^

For group B, physician raters perceived that some patients might be experiencing a greater deal of pain than they reported. For the vague-pain group (a subset of group B), physician raters perceived some pain when none was reported by the patient. Patients may appear to have suffered from their non-painful symptoms (eg, vertigo, vomiting), which may have resulted in the perception of pain by the physician. For example, the physician raters in our study may have noticed non-verbal cues from patients’ facial expressions, body language, and conversations with triage nurses and assigned a non-zero pain score.^16^ Alternatively, patients may have skipped detailed descriptions of their illnesses at ED triage until they encountered the treating physician. For example, patients presenting with nausea/vomiting might also experience headaches or abdominal pain that were not reported at triage. Regardless, ED patients may suffer from a generalized form of suffering that may not be necessarily contributed to nociceptive stimuli. Solely focusing on nociception may risk neglecting other sources of suffering, both physical (eg, vague pain) and mental (eg, stress).^30^, ^31^

Previous studies have shown that younger age, female gender, and ED diagnoses of headache and back pain were associated with higher self-reported pain scores.^32^, ^33^ Our study confirmed that younger age might be related to an overestimation of pain intensity. In addition, we also found patients with a lower triage level were also more likely to report a higher pain score. Regarding physician-level factors, previous studies have shown that female emergency physicians were more likely to administer analgesics than male physicians,^34^ while non-White physicians achieved better pain relief than White physicians with less analgesics.^35^ In this study, resident raters may tend to under-appreciate patients’ degree of pain, while senior attending physicians may be better at identifying non-verbal clues on pain intensity. These findings may again support the notion that pain assessment is the social exchange of subjective and objective meanings between the patient and clinician.^13^ As an alternative approach, recent studies have begun to test more objective measurement tools, such as automated pain assessment, by analyzing facial expressions via machine learning methods.^36^

Moreover, race and ethnicity play an important role in a physician’s perception of a patient’s pain. For example, the pain of Black Americans is often underdiagnosed and undertreated in the US, compared to that of their White counterparts.^37^, ^38^ The racial disparities may, in part, result from clinician factors. In experimental studies, participants showed more stringent thresholds for perceiving pain on Black faces, compared to White faces.^39^, ^40^ In our study, all the participants were Asian, and only 17% of them reported severe pain. The low rate of severe pain in Asians may result from cultural beliefs of Buddhism (eg, enduring pain as a way for individual growth) and increased pain tolerance.^41^, ^42^ In the US, Asian Americans showed the lowest pain prevalence across all chronic pain conditions in the National Health Interview Survey.^43^ In emergency medical services treatment in Oregon, Asian and Hispanic patients were less likely to receive a pain assessment, and all racial/ethnic patients were less likely to receive pain medications compared with White patients.^44^ Taken together, clinicians should be aware of cultural implications of pain across racially and ethnically diverse patient populations to reduce disparities in pain assessment and treatment.

## LIMITATIONS

This study has some potential limitations. First, self-reported pain scores may be limited by patients’ personal experiences, educational levels, and cognitive status.^45^ We excluded patients with psychiatric complaints or consciousness disturbance, and the results cannot be generalized to them. Patients’ verbal and non-verbal reactions might also be modified by video recording (ie, the Hawthorne effect). Second, five physician raters individually scored patients and the initial ICC for agreement was relatively low. Although investigator meetings were held to strengthen consensus on pain assessment, subtle variations may still exist. Third, we did not relate pain assessment to actual pain medications during the ED stay. This information would be helpful to elucidate the role of pain assessment in ED analgesia. Fourth, we did not edit the videos to remove the self-reported pain scores, which may have affected the physician ratings. However, the reviewers were also asked to focus on the objective clues, and we were able to detect the discrepancies between self-reported pain scores and physician perceptions of a patient’s pain. Finally, during the coronavirus 2019 pandemic, patients were asked to wear a mask in our ED, which resulted in some loss of access to facial expressions and slightly altered vocalizations.

## CONCLUSION

In this prospective videotaped study, patients’ self-reported pain scores correlate positively with the pain score provided by physicians, with only a moderate agreement. Under- and over-estimations of pain in ED patients occur in different clinical scenarios that deserve a closer look by the treating physician. Pain assessment in the ED requires a multifaceted approach considering both patient- and physician-related factors.

## Supplementary Information



## Figures and Tables

**Table 1:** Baseline clinical characteristics of emergency department patients.

Variable	(N = 279)
Age, mean (SD), year	53.3 (19.3)
Male gender, n (%)	138 (49.5)
Asian race, n (%)	279 (100)
Self-reported pain score, median (IQR)	4 (0–6)
Self-reported pain intensity, n (%)	
No pain (0)	125 (44.8)
Mild (1–3)	14 (5.0)
Moderate (4–6)	92 (33.0)
Severe (7–10)	48 (17.2)
TTAS Triage level, n (%)	
1	3 (1.1)
2	38 (13.6)
3	205 (73.5)
4	28 (10.0)
5	5 (1.8)
Triage duration, median (IQR), minutes: seconds	2:42 (2:12–3:19)
Top 5 chief complaints, n (%)	
Trauma/Injuries	40 (14.3)
Abdominal pain	30 (10.8)
Fever	23 (8.2)
Dizziness and vertigo	21 (7.5)
Chest pain	20 (7.2)

*IQR*, interquartile range; *TTAS*, Taiwan Triage and Acuity Scale.

**Table 2:** Interrater agreement of pain scoring between patient self-reports and physician ratings of patients’ pain.

	Physician ratings											

Patient self-report	Pain Score											

Pain score	0	1	2	3	4	5	6	7	8	9	10	Total
0	57	19	21	12	5	7	3	0	1	0	0	125
1	0	0	0	0	0	0	0	0	0	0	0	0
2	1	0	2	0	0	0	0	0	0	0	0	3
3	1	4	1	4	0	0	0	1	0	0	0	11
4	2	8	12	4	5	1	1	0	0	0	0	33
5	4	7	7	6	4	4	2	1	0	0	0	35
6	1	3	2	4	1	5	4	4	0	0	0	24
7	1	4	8	5	1	3	2	2	1	0	0	27
8	0	2	6	4	4	1	0	2	1	0	0	20
9	0	0	0	0	0	0	0	0	0	1	0	1
10	0	0	0	0	0	0	0	0	0	0	0	0
Total	67	47	59	39	20	21	12	10	3	1	0	279

**Table 3:** Baseline patient characteristics by agreement status of pain scoring.

	Group A^a^ N = 102		Group B^b^ N = 52	P-value
Age, mean (SD), years	49.8 (19.6)		57.4 (18.4)	0.02
Female gender, n (%)	53 (52.0)		31 (59.6)	0.37
TTAS Triage level, n (%)				0.01
1	0 (0)		2 (3.8)	
2	10 (9.8)		11 (21.2)	
3	78 (76.5)		37 (71.2)	
4	13 (12.8)		1 (1.9)	
5	1 (1.0)		1 (1.9)	
Most common chief complaint, n (%)		Most common chief complaint, n (%)		
Traumatic injuries	24 (23.5)	Dizziness/vertigo	10 (19.2)	
Abdominal pain	20 (19.6)	Fever	5 (9.6)	
Soft tissue redness/swelling	11 (10.8)	Nausea/vomiting	4 (7.7)	
Chest pain	10 (9.8)	Injuries	3 (5.8)	
Fever	4 (3.9)	General weakness	3 (5.8)	
Teeth/gum pain	4 (3.9)	Chest pain	2 (3.9)	
Urinary tract symptoms	4 (3.9)	Soft tissue redness/swelling	2 (3.9)	
Skin rash	3 (2.9)	Edema	2 (3.9)	
Flank pain	3 (2.9)	Cough	2 (3.9)	
Attending-level physician rater, n (%)	16 (15.7)		33 (63.5)	<0.001

a Physician rating is lower than patient self-report by at least 2 points.

b Physician rating is higher than patient self-report by at least 2 points.

*TTAS*, Taiwan Triage and Acuity Scale.

**Table 4:** The most common chief complaints in the vague-pain group.

Chief complaint	(N = 49)
Dizziness and vertigo, n (%)	9 (18.4)
Fever, n (%)	5 (10.2)
Nausea and vomiting, n (%)	4 (8.2)
General weakness, n (%)	3 (6.1)
Injuries, n (%)	3 (6.1)
