# Supplementary figures and images for: Pain Assessment in the Emergency Department: A Prospective Videotaped Study
Source: West J Emerg Med. 2022 Aug 28;23(5):716–23. doi: 10.5811/westjem.2022.6.55553 (PMC9541978; doi:10.5811/westjem.2022.6.55553)

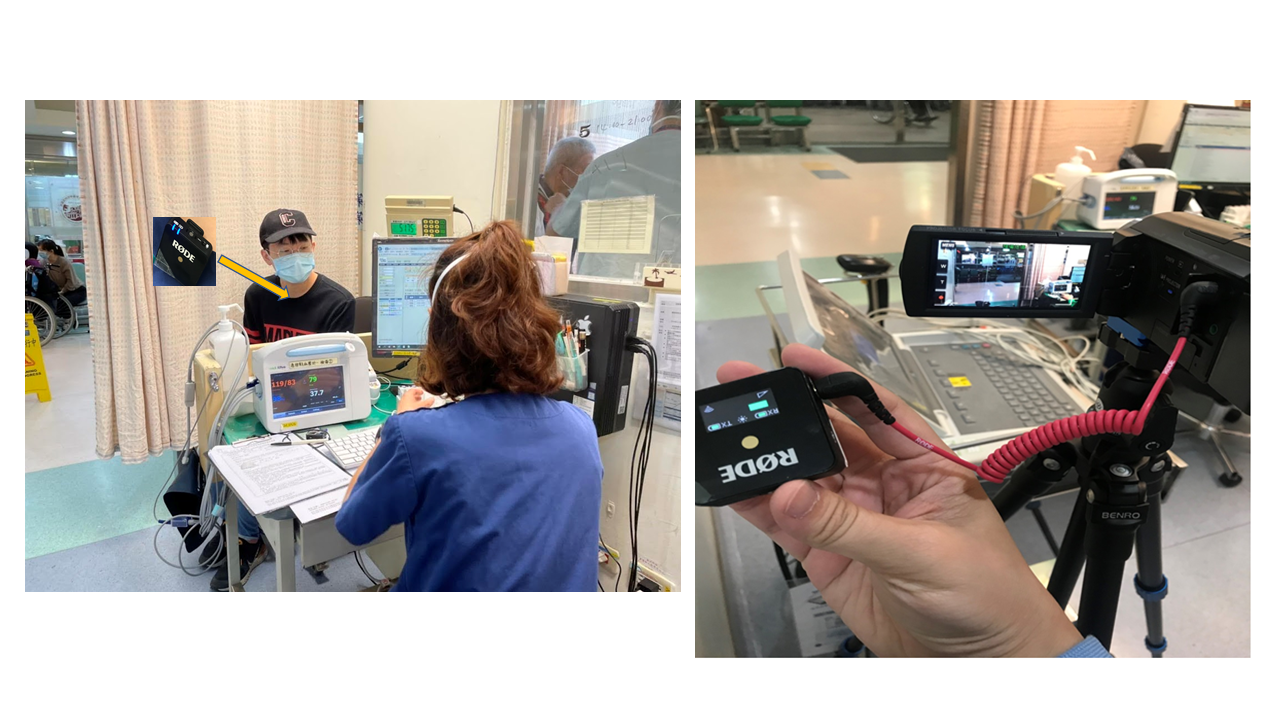

Supplement: Supplementary file 1 [file wjem-23-716-s001.tif]
